# Self-Catalyzed AlGaAs Nanowires and AlGaAs/GaAs Nanowire-Quantum Dots on Si Substrates

**DOI:** 10.1021/acs.jpcc.1c03680

**Published:** 2021-06-23

**Authors:** Giorgos Boras, Xuezhe Yu, H. Aruni Fonseka, George Davis, Anton V. Velichko, James A. Gott, Haotian Zeng, Shiyao Wu, Patrick Parkinson, Xiulai Xu, David Mowbray, Ana M. Sanchez, Huiyun Liu

**Affiliations:** † Department of Electronic and Electrical Engineering, University College London, London WC1E 7JE, United Kingdom; ‡ Department of Physics, University of Warwick, Coventry CV4 7AL, United Kingdom; § Department of Physics and Astronomy, University of Sheffield, Sheffield S3 7RH, United Kingdom; ∥ Institute of Physics, Chinese Academy of Science, Beijing 100190, China; ⊥ Department of Physics and Astronomy and the Photon Science Institute, University of Manchester, Manchester M13 9PL, United Kingdom

## Abstract

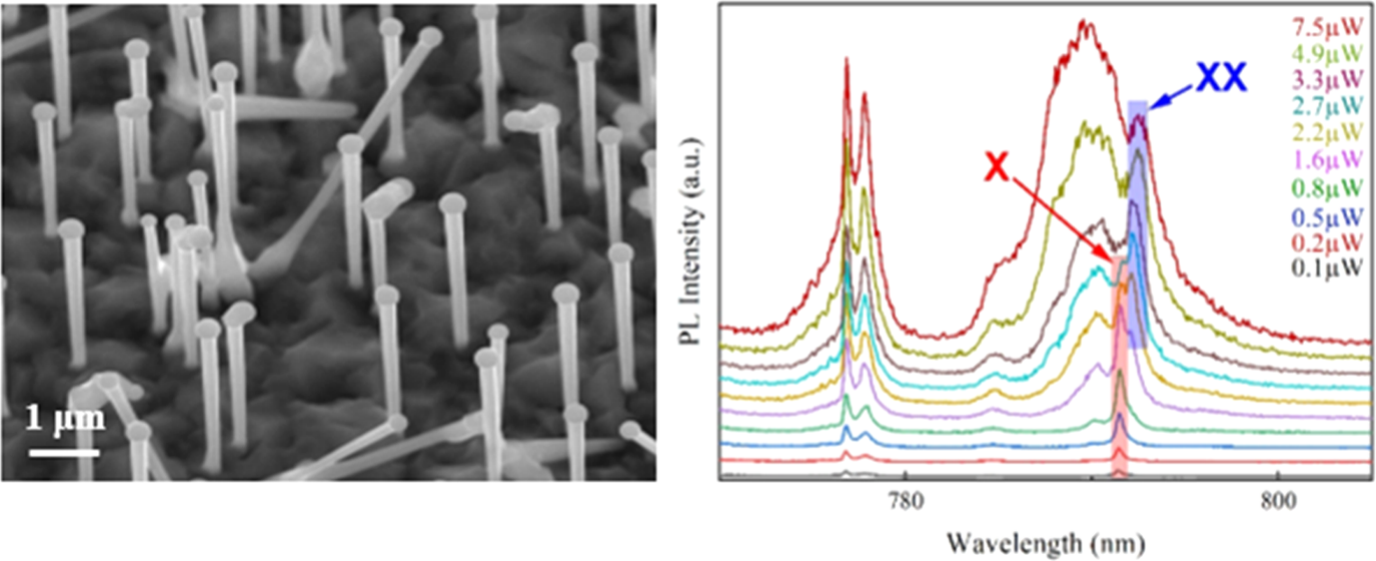

Self-catalyzed AlGaAs nanowires (NWs) and NWs with a GaAs quantum dot (QD) were monolithically grown on Si(111) substrates via solid-source molecular beam epitaxy. This growth technique is advantageous in comparison to the previously employed Au-catalyzed approach, as it removes Au contamination issues and renders the structures compatible with complementary metal–oxide–semiconductor (CMOS) technology applications. Structural studies reveal the self-formation of an Al-rich AlGaAs shell, thicker at the NW base and thinning towards the tip, with the opposite behavior observed for the NW core. Wide alloy fluctuations in the shell region are also noticed. AlGaAs NW structures with nominal Al contents of 10, 20, and 30% have strong room temperature photoluminescence, with emission in the range of 1.50–1.72 eV. Individual NWs with an embedded 4.9 nm-thick GaAs region exhibit clear QD behavior, with spatially localized emission, both exciton and biexciton recombination lines, and an exciton line width of 490 μeV at low temperature. Our results demonstrate the properties and behavior of the AlGaAs NWs and AlGaAs/GaAs NWQDs grown via the self-catalyzed approach for the first time and exhibit their potential for a range of novel applications, including nanolasers and single-photon sources.

## Introduction

Semiconductor nanowires (NWs) are promising building blocks for next-generation electronic and optoelectronic applications.^1^ In particular, III–V compound NWs have gained increasing interest due to their efficient light generation and guiding.^2,3^ Consequently, significant advances have been made in this field, with a number of direct band-gap III–V semiconductor-based NWs studied and, in several cases, directly grown on Si substrates.^4−7^

An essential step to expand the functionality range of NWs is the synthesis of ternary III–V alloys, allowing tunability of the band gap via the elemental composition. This provides an additional and controllable degree of freedom in the band-gap engineering^8^ and has been widely employed in conventional thin-film growth. Nevertheless, the successful growth of high-quality ternary NWs remains challenging, due to the sensitivity of the growth parameters and potential issues associated with inhomogeneous elemental distribution in the alloy.^8^ Despite these challenges, a variety of III–V ternary alloys have been realized.^9−16^ Among the various III–V material platforms available, AlGaAs combined with GaAs has the significant advantage of nearly identical lattice constants and a broad range of band-gap tunability, varying between 1.42 eV (GaAs) and 2.16 eV (AlAs) at room temperature (RT).^17^ The band gap is direct up to 1.98 eV, which corresponds to an Al content of ∼45%.^17^ Therefore, the planar GaAs/AlGaAs material system has led to a wide range of applications over the past few decades, including high-electron-mobility transistors,^18^ quantum well (QW) infrared detectors,^19^ and near-infrared laser diodes.^20^

AlGaAs has been successfully incorporated in GaAs NWs for a range of structures, using different growth techniques. Several groups have implemented the GaAs/AlGaAs material system in NWs for applications including lasers,^21,22^ quantum emitters,^23,24^ light-emitting diodes,^25^ and detectors.^26^ The majority of these results are limited to a GaAs core/AlGaAs shell architecture. However, the less-investigated, pure AlGaAs ternary NWs are also of great interest. For example, AlGaAs NWs could be exploited as efficient light sources on a Si chip, with a tunable emission wavelength. Furthermore, AlGaAs NWs can also act as hosts for GaAs nanostructures. For instance, radial QWs incorporated in pure AlGaAs NWs can enhance the lasing performance compared to their GaAs/AlGaAs core/shell NW counterparts,^27−30^ where the GaAs core forms a low-band-gap carrier reservoir and can also absorb lasing photons emitted by the GaAs QWs. Pure AlGaAs NWs have been exploited as hosts for quantum dots (QDs) embedded in their axis, allowing the integration of the QDs with the photonic cavity formed by the NW morphology. Specifically, AlGaAs/GaAs nanowire-quantum dots (NWQDs) with light emission in the spectral range of 600–800 nm have been reported.^31−33^

To date, published reports on AlGaAs NWs^34−38^ and AlGaAs/GaAs NWQD^31−33^ systems have used the Au-catalyzed growth technique. This significantly hinders their potential, as fast solid diffusion of Au atoms renders them incompatible with existing technology.^39^ Furthermore, it has been shown that the optoelectronic properties and material quality of self-catalyzed GaAs/AlGaAs core/shell NWs are considerably improved in comparison to their Au-catalyzed counterparts and can match the performance of planar structures.^40^ Hence, utilizing the self-catalyzed method to grow NWs on Si substrates via molecular beam epitaxy (MBE) is a critical step required for incorporating AlGaAs NWs in Si-based photonics.^41−43^ The MBE technique allows precise control of material deposition,^44^ thus enabling the realization of highly complex structures. It is noted that the focus regarding self-catalyzed ternary NWs has been mainly III–V–V alloys, including GaAsP,^9,10^ GaAsSb,^11,12^ GaNP,^13,14^ and InAsSb,^15^ in terms of growth and device application. Even though progress has been witnessed regarding the catalyst-free development of InGaAs NWs,^16^ the overall inspection of III–III–V ternary nanowires is limited compared with III–V–V nanowires, possibly due to the relatively high solubility of group III elements in the droplet, which leads to the chemical consistency of the droplet seed changing even under small alterations in the balance between group III elements, rendering the growth particularly sensitive.^8^

In this work, self-catalyzed AlGaAs NW growth on Si(111) substrates using solid-source MBE is reported. A systematic study of the morphological, structural, and optical properties of the AlGaAs NWs is presented. Initially, the impact of Al incorporation into the NWs is investigated, in terms of morphology, structure, and compositional distribution. It is shown that the NWs adopt a core/shell structure by the spontaneous formation of an AlGaAs shell, with a much higher Al concentration than the core. In addition, the shell exhibits complex alloy fluctuations. The AlGaAs NWs display strong photoluminescence (PL) emission and demonstrate band-gap tunability as a function of the Al content. Furthermore, AlGaAs NWs containing a single GaAs QD are fabricated, which exhibit spatially localized emission lines corresponding to exciton and biexciton recombination, with a low-temperature excitonic line width of 490 μeV, clearly demonstrating light emission by the quantum-confined states within the QD segment.

## Methods

AlGaAs NW samples were grown by MBE using solid Al and Ga sources and As_4_ cracker cells, on commercially available p-type Si(111) substrates. Before growth, the substrates were subjected to annealing at 660 °C for 10 min in the growth chamber to remove surface contamination. The growth was initiated by the formation of Ga-catalyzed GaAs stems, approximately 600 nm long, grown for 10 min. Next, the Al flux was introduced in order to form Ga-catalyzed AlGaAs NWs, with the nominal composition varying between 10 and 40%. The NW length ranged between 4 and 8 μm, depending on the Al composition; hence, the GaAs stems represent a very small fraction of the entire structure. The NW growth took ∼100 min, and the Ga and Al fluxes used were those needed to produce Al_x_Ga_1-x_As thin films of the same composition on GaAs(001) substrates. The Ga flux was adjusted to give a thin-film growth rate of 0.6 ML/s, as previously determined by GaAs film calibrations on GaAs(100) substrates. Unless stated otherwise, a stable As_4_ flux of 2.75 × 10^–6^ Torr was maintained, giving an As/Ga ratio of 15. Based on the information provided above, the V/III beam flux ratio of As/(Ga + Al) for the samples with 10, 20, 30, and 40% nominal Al contents is 14.053, 13.119, 12.134, and 11.075, respectively. The NWQD samples were grown under similar conditions, except that the Al supply was interrupted for seven seconds. This allowed for the deposition of Ga and As only, which led to the formation of a GaAs segment of narrower band gap in the NW axis that inherently acts as a QD. Following this stage, Al flux was resumed in order for the top part of the NW to grow. Hence, the GaAs QD was fully embedded in the nanostructures.

Scanning electron microscopy (SEM) measurements were conducted in a JEOL JSM IT-100, with the accelerating voltage set at 20 kV and at a tilt angle of 30° from the top view. Scanning transmission electron microscopy (STEM) imaging and energy-dispersive X-ray spectroscopy (EDX) analysis were performed using a doubly corrected ARM200F microscope, operating at 200 kV. The NWs were mechanically transferred to holey carbon grids for side-view analysis and the microtome sliced for the cross-section STEM analysis. Finally, PL measurements on ensembles of NWs were carried out at RT using a Nanometrics RPM2000 system with an excitation wavelength of 635 nm and a power density of approximately 500 W/cm^2^. Low-temperature (6 K) micro-PL (μPL) measurements were performed on single NWs transferred to a SiO_2_/Si substrate with a focused laser spot size of about 1 μm and excitation wavelength of 515 nm.

## Results and Discussion

**Figure 1:**
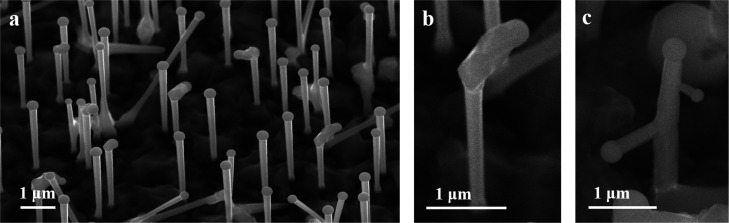
(a) SEM image with a 30° tilt angle from the top view of the AlGaAs NWs with 10% nominal Al content. (b) Kinked and (c) branched NWs are the two typical morphological variations regularly observed in these samples.

Initially, the morphology of NWs with a nominal Al content varying between 10 and 40% was investigated via SEM images. For the lowest Al concentration, the majority of the NWs adopted a vertically aligned, one-dimensional morphology (Figure 1a). However, as the Al content increases this simple morphology changes, and at 40% Al very few NWs grew perpendicular to the substrate [Figure S1a,c in the Supporting Information (SI)]. Two main morphologies of the vertical NWs are found (Figure 1b,c). The first is kinked NWs (Figure 1b), originating from the introduction of Al into the Ga droplet, which influences the preferred nucleation by changing the particle supersaturation and initiates growth along directions other than [1̅1̅1̅].^45^ The other form is branched NW growth on the AlGaAs NW side facets (Figure 1c). It is believed that the origin of this type of structure is Ga accumulation at the NW sidewalls, which causes the formation of branches via the vapor–liquid–solid (VLS) growth mode (Figure S1d in the SI). It is interesting to mention that, whilst the appearance of kinked structures is occasional, the frequency of the branched NW formation is proportional to the Al composition. Specifically, at low Al contents of 10–30%, the percentage of branched structures is low and does not exceed 15%. On the contrary, with increasing Al content (at 40%), the percentage of NWs that develop branches reaches 60%.^46^ Notably, the diameter of the NWs was also observed to increase with elevating Al content, due to the trend of this element to adhere to the sidewall facets and incorporate in the lateral shell growth. More details on this phenomenon and the analysis of these unique structures have been described in a previous report by our group.^46^ Additionally, the influence of the V/III ratio was examined, as this parameter is crucial for NW growth. Consequently, the As flux was modified in order to give V/III ratios of 15, 20, and 35. The growth is found to be very sensitive to this value, as its increase above 20 causes premature solidification of the droplets, leading to short structures with morphological deviations (Figure S1e in the SI). In what follows, we focus on fully grown NWs with the optimum morphological configuration, achieved with V/III ratios lower than 15, with the exact values that were mentioned earlier. Structural analysis and characterization were conducted for all of the acquired samples. Nevertheless, the NWs with 20% Al content were chosen as the most representative examples.

**Figure 2:**
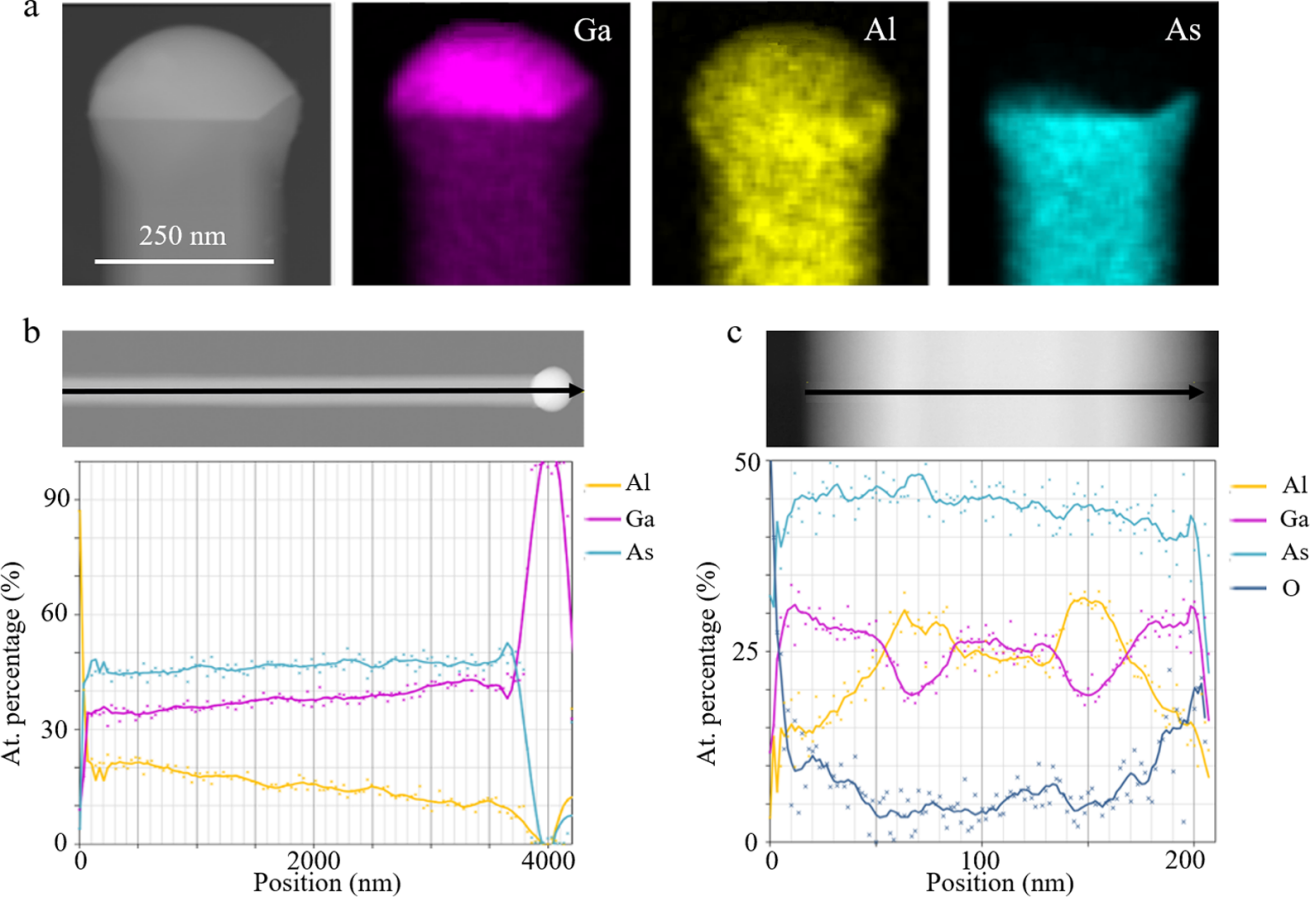
(a) Elemental distribution in an AlGaAs NW droplet region (with nominal 20% Al content) showing an Ga/Al alloy in the droplet. (b) Upper image: ADF-STEM image of a different AlGaAs NW to (a). Bottom image: Elemental-distribution profile along the NW axial direction (as indicated by the arrow in the upper image of b) showing Al and Ga inhomogeneous distributions. The droplet contains only Ga, in contrast to (a). The Ga content increases from the NW base to its tip. (c) Upper image: ADF-STEM image of the same NW of b, c, in the radial direction. Bottom image: Elemental-distribution profile in the radial direction of the NW. Whilst peaks in Al and dips in Ga are observed in the external regions, the center of the NW core exhibits a homogeneous elemental distribution for Ga and Al in the radial direction.

Structural and compositional properties of the AlGaAs NWs were studied by STEM. NWs with the Ga droplet still present, and with no kinking or branching, were selected for these measurements. Elemental-distribution EDX maps close to the tip of an AlGaAs NW with a nominal 20% Al content are shown in Figure 2a. The droplet is formed of an Al/Ga alloy, with As not observable due to its low solubility in Ga. This confirms that Al is incorporated into the Ga droplets and that AlGaAs NW formation results from the VLS growth mechanism. Nevertheless, Al is not observable in some NW droplets. A possible explanation is that Al leaves the droplet before Ga, after the termination of the group III supply.^38^Figure 2b corresponds to a different NW with the same nominal Al composition. The black arrow in the annular dark field (ADF) upper image indicates the direction of the EDX line. The elemental distribution along the nanowires, shown in the bottom image of Figure 2b, demonstrates that only Ga is found in the droplet. The elemental distribution along the NW is also found to be inhomogeneous, with Ga increasing (Al decreasing) noticeably from the base to the NW tip. The radial distribution was also addressed. The ADF upper image of Figure 2c presents the radial direction of the same AlGaAs NW. The elemental distribution is illustrated in the bottom image of Figure 2c, where distinct peaks in Al and dips in Ga are demonstrated in the external regions of the AlGaAs NW. This feature is suggestive of the core/shell configuration based on the tendency of Al to be adhered to the sidewall facets of the NWs, which will be conclusively established further on in the current report.

**Figure 3:**
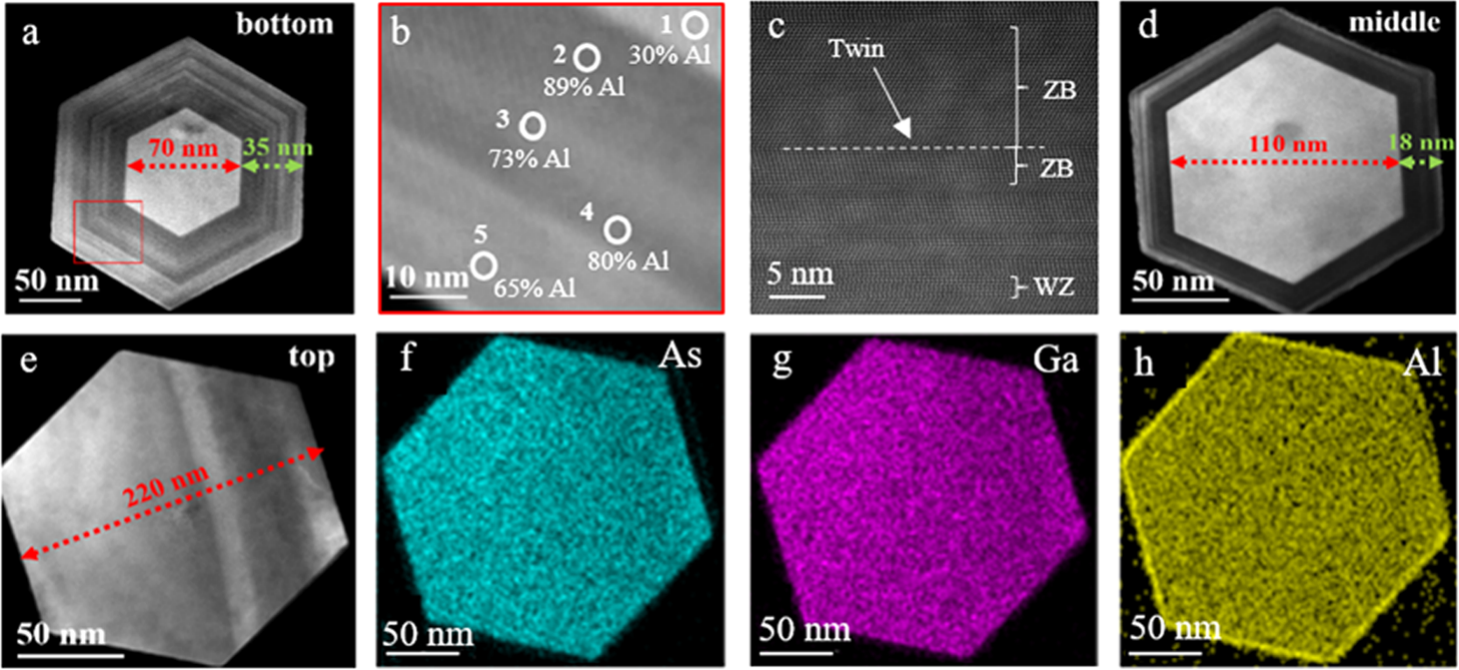
(a) ADF-STEM image of a cross section from the bottom region of an AlGaAs NW with 20% nominal Al content. The diameters of the core and shell are indicated by red and green arrows, respectively. (b) A magnified STEM image of the area enclosed in the red square in (a). The points 1–5 indicate the five positions where point-EDX measurements were performed. The Al percentage at these different positions is written below each circle. (c) Atomically resolved ADF-STEM image from a representative AlGaAs NW. Twin defects (white dashed line) and short wurtzite (WZ) insertions coexist with a predominantly ZB structure. (d, e) Representative ADF-STEM images from the middle and tip, respectively, of an AlGaAs NW with a nominal 20% composition, showing the spontaneous formation of the shell and the inverse tapering of the core NW structure. (f–h) Elemental mapping of a section close to the AlGaAs NW tip for As, Ga, and Al, respectively.

NWs were sectioned perpendicular to the growth direction using an ultramicrotome for further characterization. All sections analyzed here correspond to regions above the GaAs stems formed at the initial stages of the growth, i.e., approximately the first 600 nm of the structures. It is important to mention that these GaAs areas are not included in this study. ADF cross-sectional image, taken from the bottom part of a NW, with 20% nominal Al composition (Figure 3a) corroborates the spontaneous formation of an AlGaAs shell, with higher average Al content than the core. This is indicated by the darker contrast of the layers (∼35 nm) surrounding the brighter NW core (∼70 nm) in Figure 3a. Here, brighter regions in ADF images correspond to a higher concentration of heavier elements, which in this case is Ga. As can be observed, the region surrounding the core does not exhibit a uniform contrast, but a series of bands that correspond to different compositions. To corroborate the different Al compositions, EDX analysis was performed at four different radial points, and at the edge of the core, as indicated in Figure 3b (a magnified image of the region enclosed in the red square in Figure 3a). The measured Al values are included in the image for each point, revealing shell compositions varying from 65 to 89%. This confirms the non-uniform Al distribution as well as the higher Al content in the spontaneous shell than in the core. The Al content in the shell reaches 89% (position 2), i.e., an Al/Ga ratio of 8, much larger than values previously reported.^47^ The higher Al composition in the shell is attributed to the shorter diffusion length of Al, with a much higher activation energy for surface diffusion compared to Ga.^38,48,49^ Consequently, it is preferential for Al to be incorporated at the side facets of the structure during simultaneous vapor–solid growth, leading to the formation of the Al-rich shell. It is interesting to note that alloy fluctuations in the shell, similar to the ones observed in the current work, with alternating regions of different composition, have been previously reported and exploited for the realization of radial QWs.^27−30^ Furthermore, such alloy fluctuations have led to the self-formation of QD-like emitters and related nanostructures, which provide a novel type of nonclassical light emitter.^50,51^ Additional EDX mappings of the shell can validate the bright contrast derived from higher Al content within the shell. These measurements, along with the relevant discussion, are presented in Figure S2a–d in the SI. The origins of the observed phase separation have not been conclusively identified. It has been reported in previous works that such phenomena are attributed to the kinetics of the adatoms and more specifically to the different diffusivities of Al and Ga during the growth procedure.^47^ This has also been indicated by the fact that similar phenomena can be drastically reduced in core/shell NWs developed at lower temperatures.^47^ The above observations are in good agreement with predictions regarding the temperature-related modifications of the adatom diffusion length.^52^ However, more work is required to fully interpret the interesting formation of these alloy fluctuations.

The crystal quality of the AlGaAs NWs was studied by atomically resolved ADF. Figure 3c corresponds to a representative ADF-STEM image of the nanowire with 20% nominal Al composition along the <110> direction. Here, twin defects and occasional thin wurtzite (WZ) insertions coexist in the predominant zinc blende (ZB) crystal. An enlarged image can be found in the Supporting Information (Figure S4). Figure 3d,e shows ADF images of representative cross sections acquired at different positions along the NWs from the sample with a nominal Al composition of 20%. In combination with Figure 3a, it is seen that the thickness of the self-formed shell decreases towards the NW tip, being thickest (∼35 nm) at the base (Figure 3a), thinning (18 nm) in the middle (Figure 3d), and eventually disappearing completely in the vicinity of the tip, where only the core is present (Figure 3e). The opposite behavior occurs for the core, where the diameter is small at the base (∼70 nm) (Figure 3a) and the thickness increases towards the tip. Consequently, the total diameter of the NW increases from the bottom to the top, being roughly 105 nm at the base, 128 nm in the middle, and 220 nm at the NW tip, giving an inversely tapered shape. The increasing diameter of the NW core from the NW base to the tip is attributed to a reduction of the effective V/III ratio after introducing Al into the reactor, which contributes to the droplet volume expansion.^53^ In contrast, the thinning of the shell is attributed to the previously mentioned short diffusion length of Al, which results in Al adatoms preferentially adhering to the bottom region of the NW sidewalls, with less Al reaching the upper region of the NW. A similar explanation was proposed for an analogous observation in InGaAs NWs.^54^ A schematic representation of this mechanism is included in Section S3 of the SI. Another reason for the thickness variations is that the top part of the NW is formed at the later stages of the growth. Hence, there is not sufficient time for the shell to be developed close to the tip, as opposed to the base, where the material has been accumulating for a longer time. Eventually, the elemental mappings of the cross section close to the NW tip for As, Ga, and Al are presented in Figure 3f–h, respectively. This conclusively confirms the existence of the three elements along the radial direction of the AlGaAs NW.

**Figure 4:**
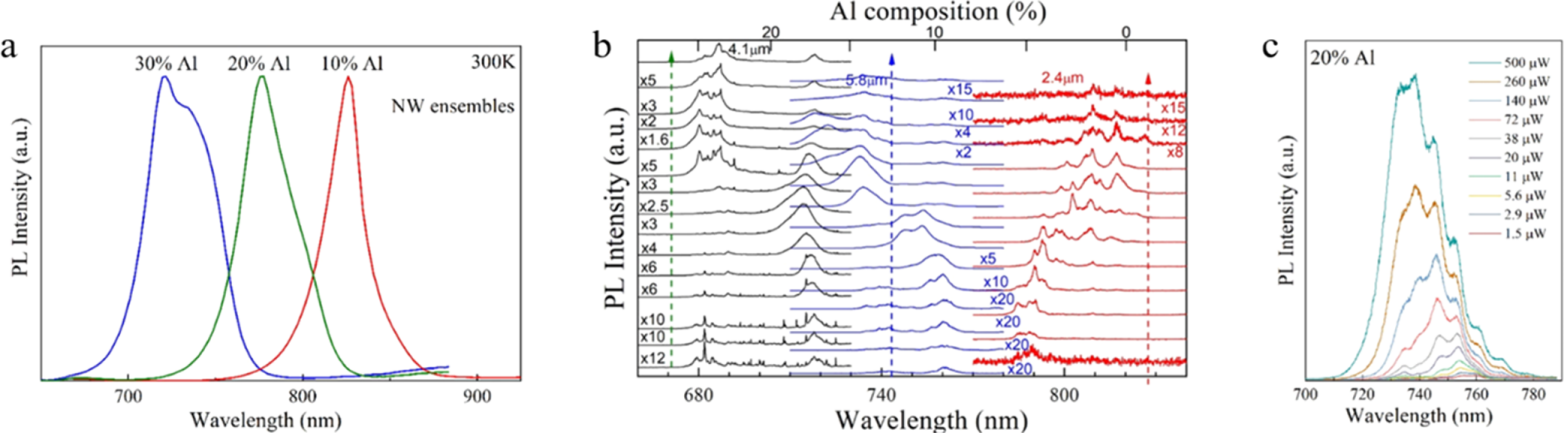
(a) RT PL spectra for AlGaAs NW ensembles with different nominal Al compositions. The blueshift of the emission confirms the tunability of the emission with ternary alloy composition. (b) Representative position-dependent μPL spectra recorded for the samples with 10% (red), 20% (blue), and 30% (black) nominal Al content, respectively. The upper axis indicates the corresponding Al composition as obtained from published values for the low-temperature band gap of zinc blende AlGaAs. (c) Power-dependent μPL spectra from a sample with nominal 20% Al content. With increasing power, the sharp emission lines merge into a broader peak, which allows the average Al composition of the NW to be determined.

RT PL was recorded for NW ensembles with 10–30% nominal Al composition and with the NWs attached to the original Si substrate. The spectra are presented in Figure 4a, where it is seen that increasing Al composition causes a blueshift of the emission, due to the increasing band-gap energy of AlGaAs. Strong RT emission is obtained for all of the three samples. The average Al composition of the structures was estimated by using the emission wavelengths of the NW ensembles and the empirical equation *E*_g_(*x*)= 1.422 + 1.2475*x*, where *x* is the Al fraction and *E*_g_ is the band-gap energy at RT.^55^ For the samples with 10, 20, and 30% nominal Al content, effective Al compositions are calculated to be 7 ± 2, 13 ± 3, and 22 ± 4%, respectively. The errors in these values are obtained by attributing the line width of the emission to the distribution of the Al content in the structures. The present observation of strong RT emission that is tunable with Al content demonstrates the potential of the AlGaAs NWs as wavelength-tunable light sources on Si. No RT emission was measured from the sample with a nominal 40% Al content, possibly due to the actual composition of this structure being close to, or even above, the value at which the band gap becomes indirect.

6 K μPL measurements were performed at positions along the axis of single NWs to probe the spatial variation of the Al composition. Figure 4b shows spectra from NWs of the three nominal compositions 10, 20, and 30%. The NWs have different lengths, as indicated in the figure. For each NW, spectra were recorded at equidistant positions along the axis. The top horizontal axis of Figure 4b is calibrated to give the Al content of AlGaAs using the band gap vs composition relationship at 6 K.^56^ The spectra exhibit a series of sharp emission lines, whose intensity and wavelength vary along the NW axis. Due to the relatively low incident laser power (6 μW) used to acquire these spectra, these lines are attributed to recombination from a low density of localized states, produced by fluctuations in the AlGaAs composition. The peaks are relatively weak in the vicinity of the NW ends. Although it is not possible to distinguish the base and tip in these measurements, the weak emission at one end most likely results from the presence of the GaAs stem at the NW base, used to initiate the NW growth. It is interesting to observe that in the case of the AlGaAs NWs with 30% nominal Al content, there are two emission bands being presented. The band at 680 nm is suspected to originate from the alloy fluctuations in the AlGaAs shell that were described earlier (Figure 3b) and have been reported to form AlGaAs QWs^27−30^ or QD-like emitters.^50,51^ This is also indicated in a previous work, where emission from the alloy fluctuations in the AlGaAs shell of core/shell NWs was traced at roughly the same spectral region.^50^ The band at 720 nm is derived from the AlGaAs NW core emission. Figure 4c shows the typical power-dependent spectra for the 20% Al sample. With increasing laser power, the multiple sharp emission lines are replaced by a broader, continuous emission, more reflective of the local average AlGaAs composition. Al compositions extracted from high-power spectra for the samples with 10, 20, and 30% nominal Al composition are 5 ± 1.2, 13 ± 2.2, and 22 ± 1.4%, respectively. These values are consistent with those extracted from the RT ensemble measurements discussed previously.

The composition values determined from EDX for the cores of the three NW samples are higher than the corresponding values determined by both the RT ensemble PL and the low-temperature μPL of single NWs. Specifically, the latter gives an effective Al composition significantly lower than the nominal value, whilst the former gives a higher Al content. To explain this difference, we consider two possible factors. First, polytypism may play a role. The polytypic nature of the crystal structure has been previously established, with the observation of thin WZ insertions of 4–6 monolayers within the dominant ZB-phase NWs (Figure 3d). An enlarged image of Figure 3d is presented in Section S4 of the SI, in order to better demonstrate the aforementioned observations. It has recently been reported that the WZ form of AlGaAs exhibits a significantly lower direct band gap than the ZB form.^57^ With both forms present in a nanostructure, carriers may diffuse into the WZ segments prior to their recombination. The resultant lower PL emission energy would hence lead to a lower calculated effective Al composition, as the ZB band-gap values were used in the calculation. Second, it is possible that PL measurements are sensitive to lower-band-gap Al-deficient regions of the NWs due to carrier diffusion and capture before recombination occurs. Structural studies show the NWs to have a complex morphology (Figure 2b,c), with regions of different Al content, and it is likely that regions with the lowest Al composition dominate the PL. A quantified comparison and discussion of these two factors are presented in Section S4 of the SI.

**Figure 5:**
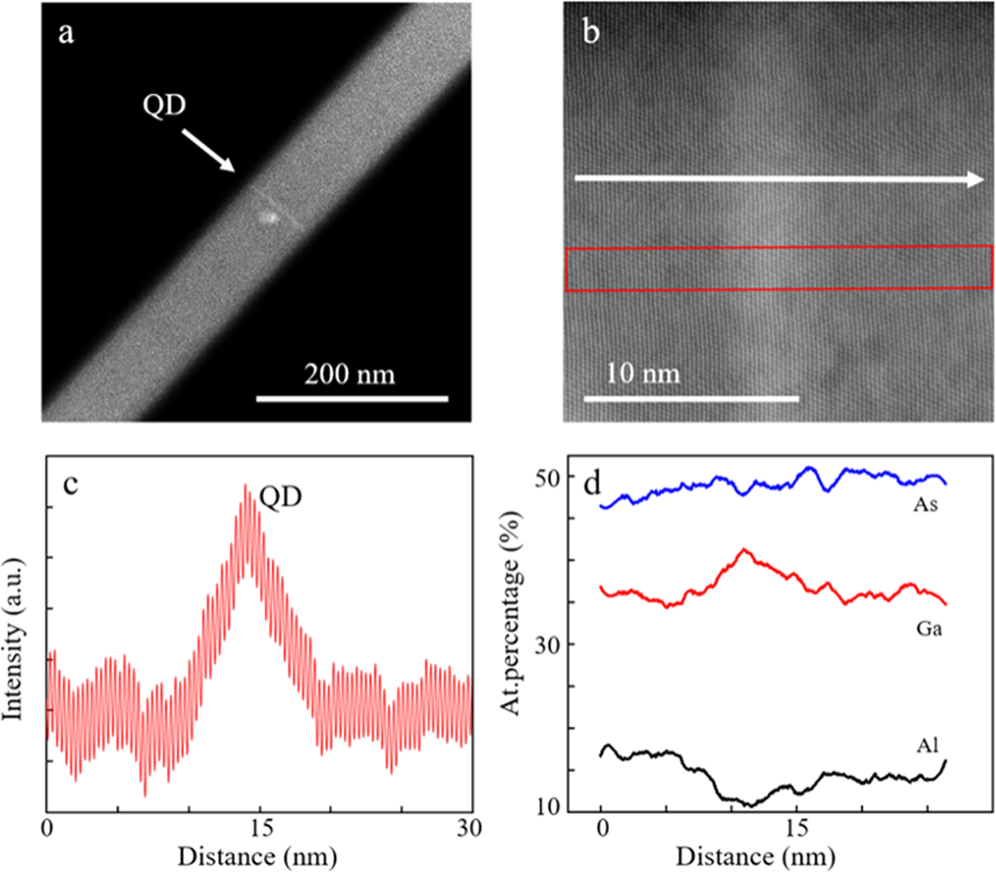
(a) ADF image of the dot-in-wire structure, where the bright region shown by the arrow corresponds to the GaAs QD. (b) Higher-magnification ADF image of the QD region. (c) Intensity profile of the area in the red box in b. The peak corresponds to the QD region, which is confirmed to be approximately 4.9 nm thick. (d) Elemental profile along the NW axis. The dip in the Al value and peak in the Ga value correspond to the GaAs QD.

After having described the AlGaAs NW properties and behavior, another interesting topic of research is the suitability of NWs for hosting GaAs dots. For this reason, AlGaAs/GaAs NWQDs were grown via MBE, with a single GaAs QD incorporated in the center of AlGaAs NWs. The QDs were deterministically embedded, axially in the AlGaAs NWs by stopping the supply of Al for 7 s, in order for the GaAs layers to be deposited. STEM analysis and structural studies of a NWQD with 20% nominal Al composition are shown in Figure 5. Figure 5a depicts an ADF image of the structures. The QD is presented as a bright band, indicating a higher Ga content, near the midpoint of the AlGaAs NW. A higher-magnification image is illustrated in Figure 5b, which reveals a QD thickness of roughly 4.9 nm and a diameter of 140 nm. To further confirm the above, the intensity profile of the area enclosed in the red box in Figure 5b is presented in Figure 5c, which indicates the QD formation at the position of the intensity peak. EDX elemental profiles along the white arrow of Figure 5b are shown in Figure 5d. The dip in the Al signal and corresponding distinct peak in Ga confirm the anticipated presence of the QD. The Al signal does not reach the expected zero value within the QD as a result of the AlGaAs shell, which is spontaneously formed around the NW and is present in the middle section of the structures (Figure 3e).

**Figure 6:**
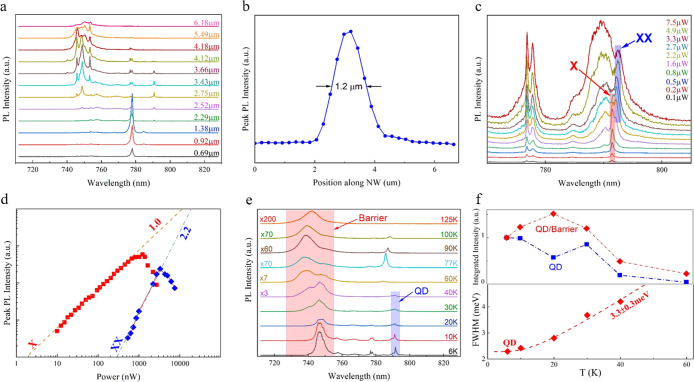
(a) Position-dependent μPL spectra measured at a low power of 50 nW. (b) QD μPL intensity vs laser position along the NW. (c) Power-dependent μPL spectra with the laser spot positioned to maximize the QD emission: exciton and biexciton peaks are indicated. (d) Laser power dependence of the exciton and biexciton emission, log–log plot. The gradients at low powers are indicated. (e) Temperature-dependent μPL spectra measured for a laser power of 1 μW; the measurement temperatures and scaling factors are shown in the figure; the QD-related emission is highlighted in blue and the main AlGaAs barrier emission in red. (f) Upper panel: Plots of the temperature dependence of the integrated intensity of the QD emission and its ratio to the barrier emission. Lower panel: Plot of the temperature dependence of the QD single-exciton emission line width.

The optical analysis on NWQDs that followed is presented in Figure 6. Specifically, Figure 6a shows low-power μPL spectra recorded as a function of the laser spot position along the axis of a NW. The lowest-energy emission at roughly 791 nm consists of a single peak, which has a relatively narrow line width of 490 μeV at a temperature of 6 K and for a low laser power of 10 nW. This emission is attributed to the GaAs dot, as it is highly spatially localized and located centrally along the NW axis. Higher-energy emission comprises two sets of peaks, grouped around 778 and 750 nm (Figure 6a). Both groups persist over a wide range of laser positions (>3–4 μm), notably larger than the laser spot size, and are hence attributed to the AlGaAs barriers. Although both groups of AlGaAs emission lines are observed for excitation on either side of the QD, a different set dominates for each side. This observation suggests that, for the current NW, the introduction of the GaAs QD results in a change in the structure of the core region of the AlGaAs NW. Further investigations are planned to clarify this effect. Nextnano simulations conducted to corroborate the experimental observations for the structure with 20% nominal Al composition^58^ give a free-particle ground-state transition energy of 1.59 eV, equivalent to 775 nm. When corrected for an exciton binding energy of ∼25 meV, typical for the GaAs–AlGaAs NWQD system,^59^ an emission wavelength of 787 nm is obtained, consistent with the experimentally observed emission peak at 791 nm. In addition, Figure 6b plots the intensity of the QD emission against laser position, which exhibits a spatial profile consistent with the size of the focused laser spot being much larger than the height of the QD. It can be distinctly shown that the PL intensity at 791 nm is stronger when the laser spot illuminates the middle of the AlGaAs NW, where the dot is located. This is an additional strong indication that the localized emission described earlier at 791 nm derives from the QD insertion.

Figure 6c shows spectra recorded as a function of laser power for the laser positioned at the maximum of the QD emission. At low power, the dot emission comprises a single line, representing single-exciton recombination. With increasing power, additional spectral lines appear, attributed to multi-exciton recombination. In Figure 6d, the power dependence of the peak intensity of the two most resolved lines is shown. The gradients of the two lines (1.0 and 2.2, respectively) indicate exciton and biexciton recombination, whilst their energy separation of 2 meV is consistent with the calculated value of biexciton binding energy of 1.5 meV in GaAs/AlGaAs QDs.^59^ Analytically, it can be seen that both resolved lines exhibit an initial increase with power. The exciton line presents a saturation and both lines decrease at higher powers. This is the expected behavior of a zero-dimensional system with increasing average carrier occupancy.

Figure 6e shows temperature-dependent μPL spectra, where the dot emission is seen to quench rapidly with increasing temperature and is fully suppressed above 60 K, indicating a low potential barrier for carrier escape from the QD. The upper panel of Figure 6f plots the integrated intensity of the QD emission and its ratio to the barrier emission. Presenting the results in terms of the ratio corrects for any possible misalignment of the sample as a result of the temperature increase. It is not possible to extract a reliable value for the activation energy of the QD PL quenching, but considering that the lowest-energy barrier emission, occurring at 778 nm, is only 24 meV above the dot-related peak, a relatively rapid decrease in the intensity of the QD emission is expected. Increasing the Al content of the AlGaAs NW surrounding the dot should result in a more stable emission. The lower panel of Figure 4f shows a plot of the temperature dependence of the line width of the QD single-exciton emission. At low temperature, the line width is much narrower, whilst it broadens with increasing temperature. This broadening is described reasonably well by a model in which carriers are scattered out of the ground state of the QD by acoustic phonons.^60^ The activation energy associated with this model is the separation between the ground state and the first excited state of the QD. The value that is extracted from the fit to the experimental data is 3 meV. Nextnano simulations give the calculated separations of the ground and first excited QD states of 1 and 0.6 meV for electrons and holes, respectively, in reasonable agreement with the extracted energy of 3 meV. It is noted that the observed discrepancy between the experimentally extracted value and the simulated values of the separation energies can be attributed to crystal imperfections related to the GaAs QD and to the non-ideally abrupt NW/QD interfaces. Details of the nextnano simulations are presented in Section S5 of the SI.

The current optical spectroscopy results for the NWQD structure show clear evidence for QD behavior at low temperatures. Emission from a spatially localized region is observed, with different excitonic recombination processes; these exhibit the expected saturation at high pump powers consistent with fully quantized states. The temperature dependence of the emission line width shows a behavior consistent with that expected for a QD. The results reveal a relatively large emission line width at low temperatures and a rapid quenching of the emission intensity with increasing temperature. The former most likely arises from fluctuating carrier occupancies in nearby defect states. Consequently, improving the crystal purity of the structures would minimize this effect and adding effective surface passivation layers would reduce the effects of surface states. Additionally, improved temperature stability would possibly result from the use of higher Al compositions in the shell of the NWs, since it is anticipated to lead to improvement of their emission properties.^61,62^ However, further works are needed to unambiguously verify the aforementioned phenomenon.

## Conclusions

In summary, MBE growth of self-catalyzed AlGaAs NWs, with and without a GaAs QD, and of varying Al content, on Si substrates has been reported. For low Al concentrations the NWs are vertically oriented, while increasing Al composition leads to complex morphologies. The spontaneous formation of an Al-rich AlGaAs shell is observed, with an inhomogeneous radial distribution of Al. The shell is thicker at the bottom of the NW and thins towards the NW tip; the opposite behavior is observed for the core. A strong RT emission and band-gap tunability from 1.50 to 1.72 eV is achieved by varying the Al composition between 10 and 30%. A GaAs/AlGaAs single dot-in-wire structure shows clear QD behavior, exhibiting spatially localized emission and both exciton and biexciton transitions. The exciton line width at 6 K is 490 μeV. These results demonstrate the potential of self-catalyzed AlGaAs NWs as the host for QD emitters for applications including single-photon sources and lasers.

## Supporting Information Available

Additional SEM images of samples with 20, 30, and 40% Al content; SEM of samples grown at higher V/III ratio; EDX mapping of the self-formed shell; schematics of the AlGaAs NW growth mechanism; calculations of the effective Al content based on PL and TEM measurements; nextnano analysis about the electronic band structure calculations (PDF)
