# Supplementary material for: Self-Catalyzed AlGaAs Nanowires and AlGaAs/GaAs Nanowire-Quantum Dots on Si Substrates
Source: J Phys Chem C Nanomater Interfaces. 2021 Jun 23;125(26):14338–47. doi: 10.1021/acs.jpcc.1c03680 (PMC8279736; doi:10.1021/acs.jpcc.1c03680)
Supplement: Supplementary file 1 — jp1c03680_si_001.pdf [file jp1c03680_si_001.pdf]

Self-Catalyzed AlGaAs Nanowires and AlGaAs/GaAs Nanowire-Quantum Dots on Si Substrates

Giorgos Boras,[†] Xuezhe Yu,[†] H. Aruni Fonseka,[‡] George Davis,[§] Anton V. Velichko,[§] James A. Gott,[‡] Haotian Zeng,[†] Shiyao Wu,[‡] Patrick Parkinson,[└] Xiulai Xu,[‡] David Mowbray,[§] Ana M. Sanchez[‡] and Huiyun Liu[†]

[†] Department of Electronic and Electrical Engineering, University College London, London WC1E 7JE, United Kingdom

[‡] Department of Physics, University of Warwick, Coventry CV4 7AL, United Kingdom

[§] Department of Physics and Astronomy, University of Sheffield, Sheffield S3 7RH, United Kingdom

[‡] Institute of Physics, Chinese Academy of Science, Beijing 100190, China

[└] Department of Physics and Astronomy and the Photon Science Institute, University of Manchester, Manchester M13 9PL, United Kingdom

1) Scanning Electron Microscopy Analysis of the Samples

The morphological investigation of the AlGaAs nanowire (NW) samples was performed using Scanning Electron Microscopy (SEM) on structures with different nominal Al compositions. In the main text, the SEM of NWs with 10% nominal Al content was presented, which exhibited perpendicular, one-dimensional morphology, typical for ternary NWs. Here, SEM images of the NWs with 20%, 30% and 40% nominal Al composition are shown in Figs. S1a-c, respectively. The increase in Al leads to significant variations in the morphology of the structures. For 20% and 30% nominal Al concentration, the NWs are still perpendicular to the Si substrate (Figs. S1a-b). The spherical droplet close to their tips confirms growth by the vapour-liquid-solid (VLS) mode. In contrast, $Al_{0.4}Ga_{0.6}As$ NWs exhibit significant differences, with the structures exhibiting severe kinking and branching that distorts their (111) orientation. Consequently, only a minority of these NWs remain vertically aligned on the substrates, with the majority of the NWs demonstrating two main morphological variations, kinking and branching. The dimensions of the NWs also vary with Al content. Increasing the Al content results in a continuous increase in the NW diameter and a corresponding reduction in their length. The spontaneous core/shell formation that is discussed in detail in the main paper could explain this observation. Since Al tends to incorporate preferentially at the external, lower regions of the NWs, causing the formation of the Al-rich shell, an increase in the Al composition should result in an

enhancement of the NW diameter and a reduction of the NW length. Another morphological variation of the AlGaAs NWs is the formation of branches to the main stem. The SEM image of Figs. S1d, shows a characteristic example of a branched NW, with two branches nucleated on the sidewall facets. The NW has a nominal 40% Al concentration, which is the highest Al content used for the NW growth. A detailed analysis of these intriguing characteristics is presented elsewhere.[1]

The effect of the III/V ratio, which is an important parameter for NW growth, was also studied. By varying the As flux, V/III ratios of 15, 20 and 35 were obtained. The growth is found to be particularly sensitive to this parameter, as an enhanced group V flux causes a more rapid consumption of the alloy droplet. As a result, the droplets solidify sooner and the growth is terminated prematurely, this causes the NWs to be very short, or not to grow at all (Fig. S1e).

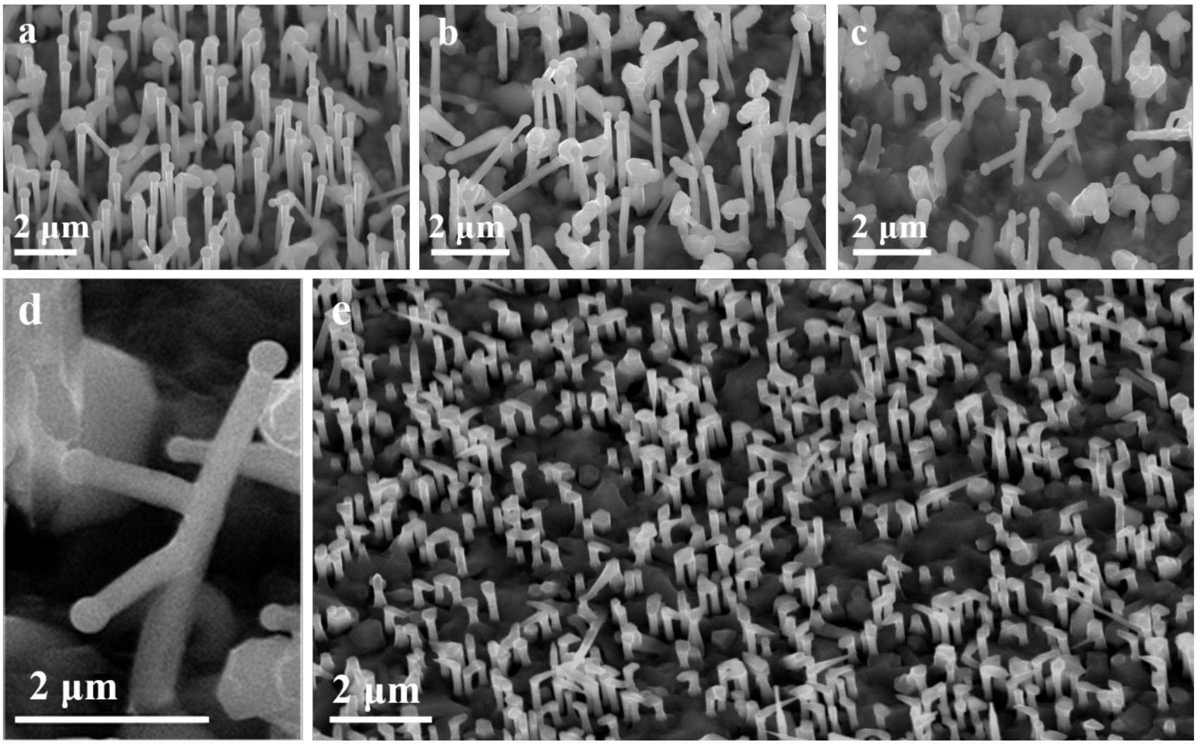

Figure S1: a-c) SEM images of ternary AlGaAs NWs with nominal 20%, 30% and 40% composition of Al, respectively. The average NW diameters for images a-c were 250 nm, 300 nm and 400 nm, while the average NW lengths were 6 µm, 5 µm and 4 µm, respectively. d) High magnification SEM image of a typical branched NW with two branches. e) SEM images of the samples grown under an increased V/III ratio of 20. The growth is terminated prematurely due to the solidification of the droplet.

2) Inhomogeneous elemental distributions in the AlGaAs shell

Spontaneously formed AlGaAs shell was studied using energy dispersive x-ray (EDX) mapping. In Fig. S2a an AlGaAs NW structure, with nominal 20% Al content, is presented, showing a high magnification cross-sectional ADF STEM image of the shell, where conspicuous contrast variations appear. The origins of these variations are compositional fluctuations in the elemental distribution of the AlGaAs. Fig. S2b illustrates an image, where the individual maps of Ga (Fig. S2c) and Al (Fig. S2d) are overlapped, with purple regions being Ga-rich and yellow regions being Al-rich. This image confirms the varying Al and Ga compositions throughout the shell, forming a complex compositional variation in this part of the NW. These results demonstrate that the NW shell comprises a highly inhomogeneous distribution of Al and Ga.

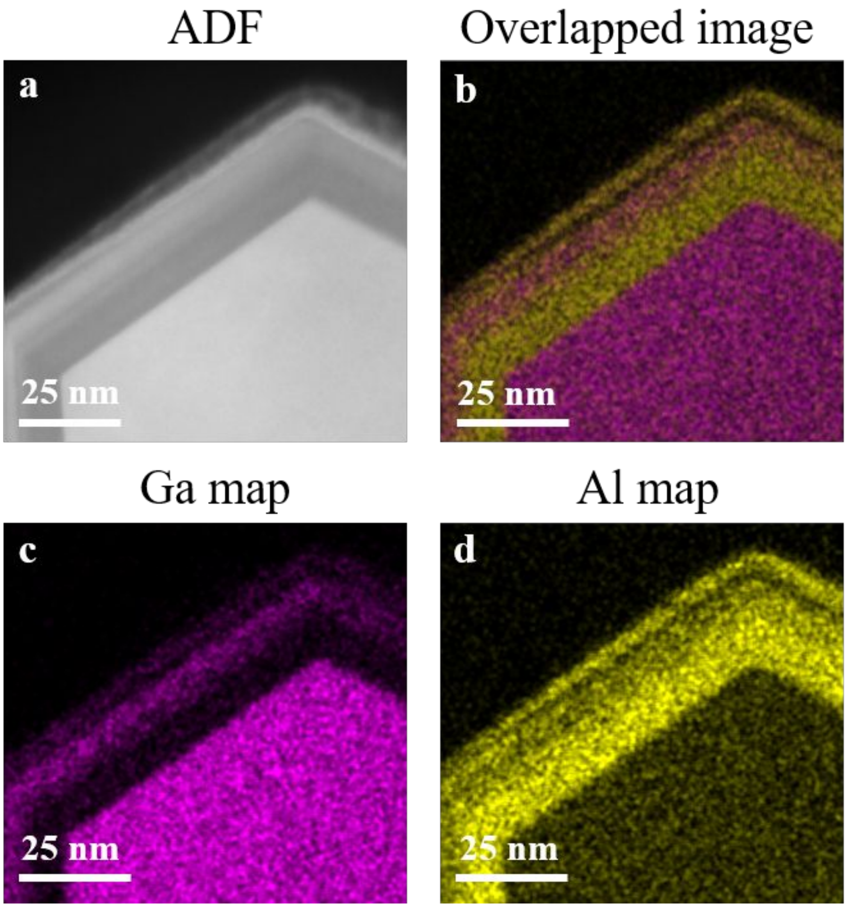

Figure S2: a) High magnification ADF image of the shell, depicting the contrast variation. b) Elemental map of the same image as that in (a), where purple and yellow regions indicate Ga -rich and Al -rich regions respectively. c-d) Individual EDX maps of Ga and Al, respectively, exhibiting both Al and Ga-rich regions in the NW shell.`

3) Schematics of the AlGaAs NW growth mechanism

The growth mechanism that results in the formation of the AlGaAs NWs is largely determined by the intrinsic diffusion lengths of Al and Ga. Arriving adatoms can be incorporated either via direct impingement on the catalyst droplet or via diffusion along the NW sidewalls. For the latter, the intrinsic diffusion lengths of Al and Ga are crucial in determining the morphology of the NW, including the spontaneous formation of the AlGaAs shell and its varying thickness. The longer diffusion length for Ga atoms makes it more likely that they diffuse to the NW tip and reach the Al-Ga droplet, from where they contribute to the axial elongation of the structures via the VLS growth mode. This property accounts for the higher percentage of Ga present in the core. In contrast, the short Al diffusion length makes it less likely for Al adatoms to reach the droplet and they are preferentially incorporated at the sidewall facets via the lateral VS growth mechanism.

The above phenomena are shown in the schematic representation of Figs. S3. In Fig. S3a, the initial elongation of the NW is attributed either to direct impingement of the adatoms on the droplet or to diffusion on the substrate and then NW sidewalls. As the NW length increases (Fig. S3b), the lower diffusion length of Al leads to less Al adatoms reaching the droplet. Consequently, the VLS grown core is Ga-rich. At the final stages of the growth (Fig. S3c), the length of the NW is such that the vast majority of Al adatoms do not reach the droplet and are preferentially incorporated at the NW sidewalls via the lateral VS growth mechanism. In addition, as the length of the NW increases during growth, the short diffusion length of the Al adatoms causes them to preferentially adhere to the sidewalls at the base of the NW rather than closer to tip. Consequently, less Al reaches the upper regions of the NW, which are Ga-rich. In the main paper it is shown that the upper region of the NW is core-only (see TEM analysis presented in Figs. 3). It is noted that the core has an inversely tapered shape. On the contrary, the shell grows thinner from bottom to top as corroborated by the ADF images of Figs. 3 in the main paper.

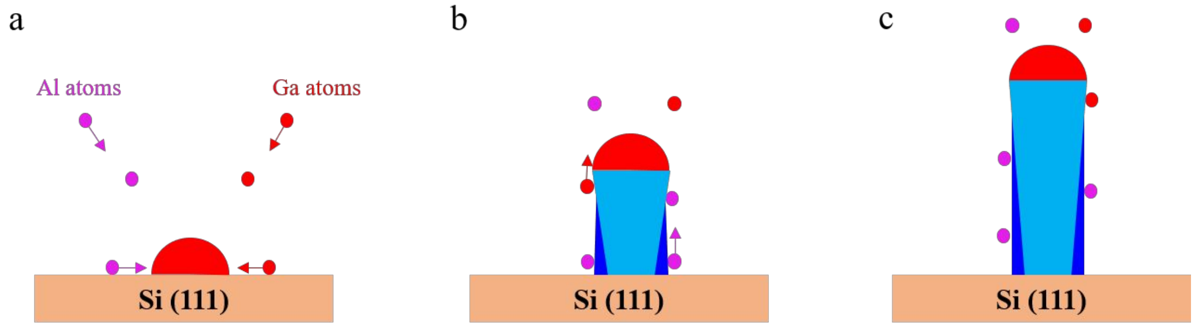

Fig. S3: a-c) Growth mechanism for the AlGaAs NWs, depicting the formation of the Al-rich AlGaAs shell with decreasing thickness from bottom to top.

4) Compositions calculated using TEM and PL analysis

In the main text, an explanation was formulated to explain the discrepancy between the composition of the alloy based on EDX measurements and the PL data. The predominantly ZB crystal exhibits WZ insertions, which in turn lead to a lower bandgap of these regions when compared to the ZB sections. For clarity, an enlarged image of Fig. 2g from the main text is presented below (Fig. S4). The structure contains significant twinning density, with three representative examples being marked by the dashed white lines. Furthermore, two examples of ZB segments are noted, along with a WZ insertion of 6 MLs thickness, corresponding to roughly 3.4 nm. In addition, a quantification of the measured and predicted values of Al content is presented. Specifically, in Table S1, the average Al contents derived via different methods are presented. Values measured via EDX are 13.5±2.3%, 29±5% and 39±5% for the samples with nominal 10%, 20% and 30% Al composition, respectively. Hence, the effective Al percentage is higher than the nominal value for all three samples. In contrast, the Al compositions obtained from PL measurements, assuming emission is from ZB material, are 7±2%, 13±3% and 22±4% from RT PL and 5±1.2%, 13±2.2% and 22±1.4% from 6 K μPL measurements. Thus, it is clear that μPL and RT PL give a consistently lower Al content, in all cases being lower than the nominal Al content of the NWs.

Table S2 also compares the predicted Al compositions assuming that the recombination occurs in either ZB or WZ AlGaAs.[2-3] There is much better agreement with the EDX determined values if the recombination comes from the WZ crystal phase. However, it is not possible to unambiguously affirm this mechanism as the WZ insertions are typically only ~6 monolayers thick (Fig. 2g). The second possible mechanism proposed to explain the discrepancy is that the PL is highly sensitive to small regions of lower bandgap material, which reflect a lower Al content. Since the dimensions of the NW are small and considering the high level of compositional variation (Figs. 2b-c of the main text), it is likely that carriers generated in regions of higher bandgap will diffuse and be captured into lower bandgap regions before recombining. In contrast, EDX data reflects the average value of the Al content in the measured volume, which is expected to be larger than the nominal value due to the higher incorporation rate of Al compared to Ga at the growth temperature used. These two proposed mechanisms could explain the discrepancy between the EDX and PL determined compositions, and a combination of both mechanisms is likely.

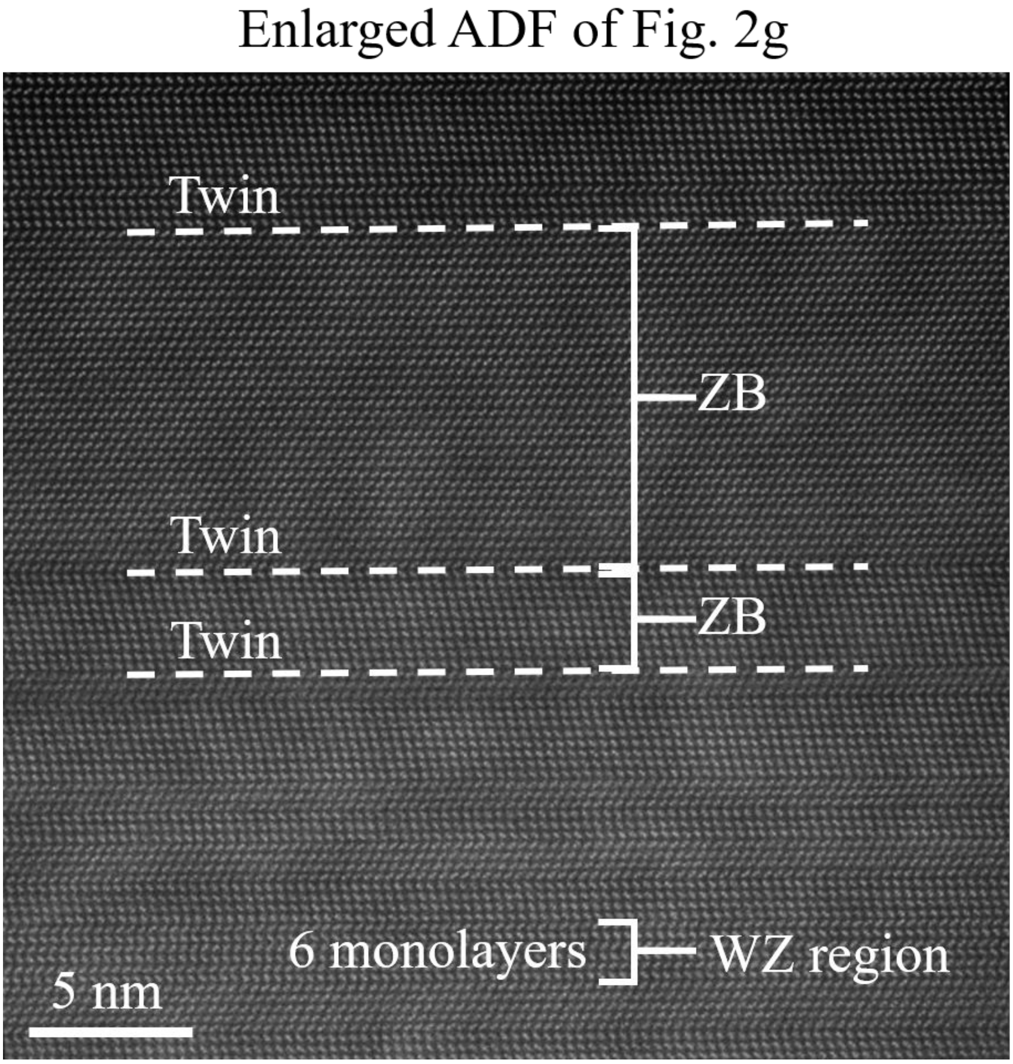

Figure S4: Enlarged ADF image from Fig. 2g. Twins indicated by the dashed white lines, in ZB regions were observed. A 6 ML thick WZ insertion was also present in this area.

Nominal Al %	EDX Al %		PL RT Al %	μPL 6 K Al %
	Range	Mean Value and Standard Deviation		
10	11-18	13.5±2.3	7±2	5±1.2
20	17-48	29±5	13±3	13±2.2
30	32-49	39±5	22±4	22±1.4

Table S1: Table summarizing the measured Al % via EDX and the calculated values based on RT PL and 6 K μPL data for the samples with nominal 10%, 20% and 30% Al composition, respectively.

Nominal Al %	ZB Al % (µPL-based)	WZ Al % (µPL-based)
10	5±1.2	9.65±0.05
20	13±2.2	20.2±2.3
30	22±1.4	35.8±0.8

Table S2: Table summarizing the calculated Al % based on the µPL spectra, assuming ZB or WZ crystal phases, respectively.

5) Electronic band structure calculations

To support the experimental results, the electronic band structure of the NWQD sample was calculated using nextnano software,[4-5] with selected results presented in Figs. S5. The cross-sectional geometry of the simulated structure is shown above and below the QD (Fig.S5a) and through the QD (Fig.S5b). For the NW core region, an effective Al content of 20% was assumed, as deduced from the PL measurements, whilst the Al composition of the shell was taken as 70%. A minimised strain model was employed. The width of the simulated hexagonal QD was assumed to be 110 nm normal to the NW axis, with a height 5 nm along this axis. Fig.S5c shows calculated conduction and valence band edges along the NW axis along with the energies of the lowest QD electron and hole confined states for non-interacting particles. The energy separation of the ground hole and electron states is 1.599 eV. Subtracting the exciton binding energy, which for a GaAs/AlGaAs NWQD of the current dimensions is around 25 meV,[6] gives a calculated exciton emission energy of 1.574 eV. This corresponds to an emission wavelength of 787 nm, very close to the experimentally observed value of 791 nm. The separations of the ground and first excited QD states are calculated to be 1 meV for electrons and 0.6 meV for holes, respectively. These values are in reasonable agreement with the 3 meV activation energy deduced from fitting the experimental temperature dependence of the exciton linewidth with a model based on exciton scattering by acoustic phonons.[7]

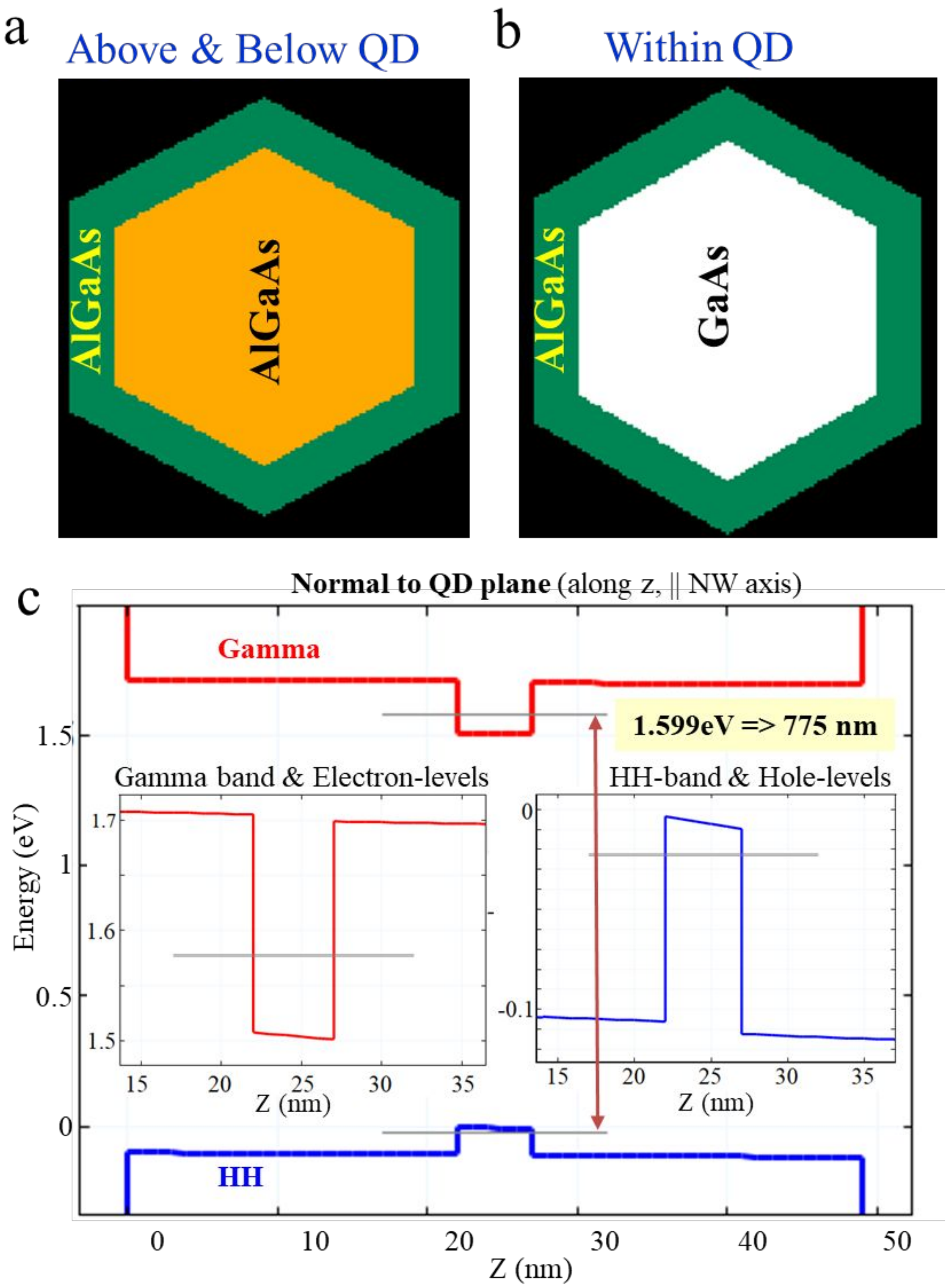

Figure S5: 8-band kp nextnano simulations: a-b) 2D slices of the simulated structures above and below the QD (Fig. S5a) and through the QD (Fig. S5b). The simulated NWs have a core with 20% Al and a shell with 70% Al. c) band edge profiles along the central axis of the NW and energies of the lowest electron and hole confined QD states. The insets show the ground state electron and hole levels on an expanded scale.

References

1) Boras, G.; Yu, X.; Fonseka, H. A.; Zhang, D.; Zeng, H.; Sanchez, A.; Liu, H. Checked-patterned elemental distribution in AlGaAs NW branches via vapor-liquid-solid growth. *Nanoscale* **2020**, 12, 15711-15720

2) Leandro, L; Reznik, R.; Clement, J. D.; Repan, J.; Reynolds, M.; Ubyivovk, E. V.; Shtrom, I. V.; Cirlin, G.; Akopian, N. Wurtzite AlGaAs Nanowires. *Sci. Rep.* **2020**, 10, 735

3) Bechstedt, F.; Belabbes, A. Structure, energetics and electronic states of III-V compound polytypes. *J. Phys. Condens. Matter* **2013**, 25, 273201

4) Fonseka, H. A.; Velichko, A. V.; Zhang, Y.; Gott, J. A.; Davis, G. D.; Beanland, R.; Liu, H.; Mowbray, D. J.; Sanchez, A. M. Self-Formed Quantum Wires and Dots in GaAsP-GaAsP Core-Shell Nanowires. Nano Lett. **2019**, *19*(6), 4158-41655

5) Birner, S.; Zibold, T.; Andlauer, T.; Kubis, T.; Sabathil, M.; Trellakis, A.; Vogl, P. nextnano: General Purpose 3-D Simulations. *IEEE Trans. Electron Devices* **2007**, 54, 2137-2142

6) Trabelsi, Z.; Yahyaoui, M.; Boujdaria, K.; Chamarro, M.; Testelin, C. Excitonic complexes in strain-free and highly symmetric GaAs quantum dots fabricated by filling of self-assembled nanoholes. *J. Appl. Phys.* **2017**, 21, 245702

7) Gammon, D.; Snow, E. S.; Shanabrook, B. V.; Katzer, D. S.; Park, D. Homogeneous Linewidths in the Optical Spectrum of a Single Gallium Arsenide Quantum Dot. *Science* **1996**, 273, 87-90
